# Useful surrogates of soil texture for plant ecologists from airborne gamma‐ray detection

**DOI:** 10.1002/ece3.3417

**Published:** 2018-01-16

**Authors:** Cassia F. Read, David H. Duncan, Chiu Yee Catherine Ho, Matt White, Peter A. Vesk

**Affiliations:** ^1^ School of BioSciences University of Melbourne Parkville VIC Australia; ^2^ Department of Environment, Land, Water and Planning Arthur Rylah Institute for Environmental Research Heidelberg VIC Australia

**Keywords:** boosted regression tree models, clay, field estimation, Gamma radiometric data, particle size analysis, potassium, remote sensing, sand, soil texture, thorium

## Abstract

Plant ecologists require spatial information on functional soil properties but are often faced with soil classifications that are not directly interpretable or useful for statistical models. Sand and clay content are important soil properties because they indicate soil water‐holding capacity and nutrient content, yet these data are not available for much of the landscape. Remotely sensed soil radiometric data offer promise for developing statistical models of functional soil properties applicable over large areas. Here, we build models linking radiometric data for an area of 40,000 km^2^ with soil physicochemical data collected over a period of 30 years and demonstrate a strong relationship between gamma radiometric potassium (^40^K), thorium (²³²Th), and soil sand and clay content. Our models showed predictive performance of 43% with internal cross‐validation (to held‐out data) and ~30% for external validation to an independent test dataset. This work contributes to broader availability and uptake of remote sensing products for explaining patterns in plant distribution and performance across landscapes.

## 1 INTRODUCTION

Plant ecologists require accurate, spatially explicit environmental data to explain and predict vegetation pattern and process with quantitative models. Among environmental variables, edaphic variables are perhaps the most important for understanding plant ecology. Soil characteristics, often generalized as texture, nutrient availability, and influence on water, are critical in limiting plant species distributions (Diekmann, Michaelis, & Pannek, 2015) through influence on colonization and growth (Laliberté et al., 2012), and recovery and degradation patterns (Baer, Meyer, Bach, Klopf, & Six, 2010). Therefore, soil data are invaluable for developing spatial models in plant ecology. But, outside of agricultural land, there is a scarcity of accurate soil data at fine grain over large extents, particularly in natural and seminatural contexts. Field data (from point estimates) are often extensively interpolated across the landscape (Taylor, Smettem, Pracilio, & Verboom, 2002) and groundtruthing is not always possible because soil data are costly and time‐consuming to collect (Diekmann et al., 2015; Pracilio, Adams, Smettem, & Harper, 2006). Further, while soil maps generally provide categorical data on soil properties such as soil class (Moonjun, Shrestha, Jetten, & van Ruitenbeek, 2017; IUSS Working Group WRB, 2015), continuous data such as percent clay or sand content are seldom available. Continuous variables are desirable because they allow for flexibility in choice of modeling method and for more precise and generalizable models.

Recent research suggests a remote sensing alternative to field sampling and soil analysis (Dent, MacMillan, Mayr, Chapman, & Berch, 2013). Soil radiometric data extracted from airborne gamma‐ray spectrometry have potential to be a cheaper and more precise method for continuous and quantitative soil mapping across large landscapes (McBratney, Mendonca Santos, & Minasny, 2003; Wong & Harper, 1999). Gamma‐ray radiation, emitted from the natural decay of radioactive elements in the earth's surface, is passively sensed by radiometers mounted on aircraft (Bierwith, 1996) or from portable ground‐based units for small‐scale observation (Viscarra Rossel, McBratney, & Minasny, 2010). The intensity of gamma‐ray emissions is influenced by the bedrock and the processes of weathering and deposition (IUSS Working Group WRB, 2015) and is proportional to the relative abundance of three elements: potassium (^40^K), thorium (²³²Th), and uranium (²³^8^U). The majority of emissions detected by spectrometers are restricted to the top 30 cm of the ground surface (Taylor et al., 2002), with radiation signals attenuating with increased soil bulk density (Cook, Corner, Groves, & Grealish, 1996) and soil moisture (Carroll, 1981). To date, airborne gamma radiometric data have been applied widely in agriculture and geology, for precision fertilizer application (Pracilio et al., 2006; Wong & Harper, 1999), determining soil moisture content (Carroll, 1981) and thickness of surface litter (Aznar, Paucar‐Munoz, Richer‐Laflèche, & Bégin, 2010), detection of different rock types (Cook et al., 1996), and ore exploration in the mining industry (Bierwith, 1996). Many recent studies have linked variation in ground‐based gamma‐ray emissions with soil physical properties, bedrock, and chemistry (e.g., Dierke & Werban, 2013; Priori, Bianconi, & Costantini, 2014; Coulouma, Caner, Loonstra, & Lagacherie, 2016; Heggemann et al. 2017), demonstrating the considerable promise of and limitations of the approach. Plant ecologists are unlikely to be able to access ground‐sensed radiometric data at the spatial scales of interest, but the potential of airborne radiometric data to represent landscape variation in soil physical properties is still poorly understood (see Cattle, Meakin, Ruszkowski, & Cameron, 2003; Dent et al., 2013; Grundy et al., 2015; Rouze, Morgan, & McBratney, 2017; Taylor et al., 2002).

White et al. (2003) observed that soil gamma radiometric K and Th signals, together with annual rainfall data, were correlated with vegetation patterns in the expansive alluvial plains and aeolian dune fields of northwestern Victoria, Australia. White et al. proposed that radiometric data were potentially a surrogate for soil texture in predictive models of vegetation composition and structure. Soil texture is a measure of the relative proportion of sand, silt, and clay particles in the soil (McDonald, Isbell, Speight, Walker, & Hopkins, 1990). It influences plant growth directly by affecting root penetration (Bengough, 2005) and indirectly through its effect on soil moisture availability (Coffin & Lauenroth, 2011; Fernandez‐Illescas, Porporato, & Laio, 2001) and availability of critical nutrients such as nitrogen (Fernandez‐Illescas et al., 2001; Pugnaire, Armas, & Valladares, 2004). Around the same time as White et al.'s study, in 10 physiographic case studies of similar physiographic formations, Cattle et al. (2003) found that topsoils with strong clay content were distinguishable from sandy soils using radiometric Th and K.

Since the publication by White et al. (2003), plant ecologists have identified several relationships between vegetation structure and radiometric signal. For instance, gamma Th and K data were related to *Eucalyptus microcarpa* seed production (Vesk, Davidson, & Chee, 2010), biological crust cover (Read, Duncan, Vesk, & Elith, 2008, 2011), and nutrient contents (N, P, and K) of soils in remnant woodlands (Duncan, Dorrough, White, & Moxham, 2007).

Soil scientists (e.g., Cattle et al., 2003) have provided rich mechanistic insight into how the interplay between erosion and deposition sources may combine to influence gamma radiometric signal at local scales, but plant ecologists are eager to know to what extent radiometric data may be used as a surrogate for soil texture or other soil properties in modeling applications. The objectives of this study therefore were to better understand the relationship at landscape scales between soil signals of ɣTh and ɣK and soil properties. Specifically, we used boosted regression tree models to (i) explore explanatory relationships between soil physical properties (including soil texture, electrical conductivity, and soil pH) and gamma spectrometry data for a region of 40,000 km^2^; and (ii) test the usefulness of ɣTh and ɣK to predict soil texture to an independent dataset from the same region.

## 2 MATERIALS AND METHODS

We developed explanatory and predictive models of the relationships between soil properties and gamma radiometric data using two independent datasets.

### 2.1 Study region

**Figure 1:**
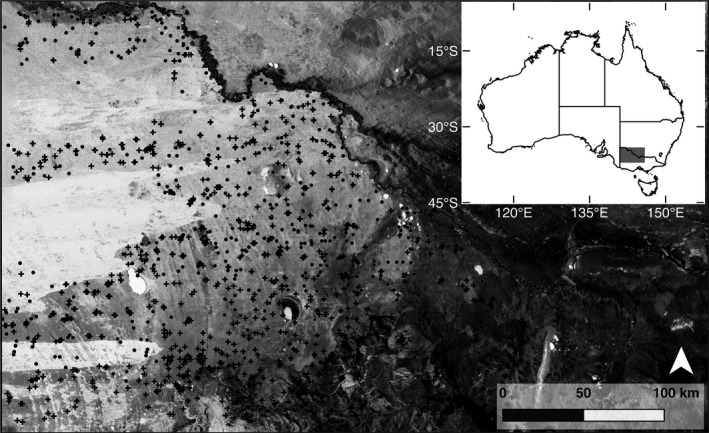
Map of the study area in Australia (inset), featuring a grayscale image of radiometric Th (darker shade = greater emission), with the locations of soil pits (training dataset, crosses), and the test dataset (circles) superimposed

Data for this study were collected in northwest Victoria, Australia (34.0°–37.0°S and 141.0°–144.4°E; Figure 1), across a region of some 40,000 km^2^. The region is relatively flat with mean elevation of less than 200 m above sea level. The geomorphology of the region is characterized by unconsolidated to sublithic surficial materials deposited in a series of episodic aeolian, lacustrine, and fluvial processes (Bowler & Magee, 1978). Extensive siliceous and calcareous dunes and parna mantle much of the landscape apart from more restricted regions of active alluvial deposition or groundwater‐controlled deflation (Pell, Chivas, & Williams, 2001; White et al., 2003). These overlapping phases of deposition and the disparate origins of the deposited materials have resulted in a landscape with contrasting soil properties, from fine‐textured fertile clays to coarse‐textured infertile sands (White et al., 2003). Importantly, exposures of bedrock and waterlogged soil, which may interfere with relationships between radiometric data and soil properties, are restricted in extent.

The region has a Mediterranean climate and is semiarid with prolonged periods of low rainfall (White, 2006). Long‐term average monthly temperature ranges from 22.4°C in summer to 8.5°C in winter. At Beulah (a location in the center of the study area), average annual rainfall is approximately 370 mm, and most precipitation occurs between May and October (Bureau of Meteorology 2017).

### 2.2 Data

Two soil datasets were available for modeling, which varied slightly in collection methodology and data available. A primary dataset was used for model training and development; a secondary dataset was used for model validation (testing).

The primary dataset was extracted from the Victorian Government** **Department of Economic Development, Jobs, Transport and Resources’ Victorian Soil Information System (VSIS; Hunter, Williams, & Robinson, 2010). The VSIS database collates soil data on 145 soil variables for approximately 1,500 sites within the study region.

Soil samples were collected from soil pits, existing vertical exposures, soil core, or auger boring between 1967 and 1997. We extracted data for 925 sites and 20 soil variables and hereafter call this the “VSIS training dataset” (variables are detailed in Table 2). Criteria for including data were as follows: Variables must be ordinal or continuous; variables must have data for >1,000 sites; variables must not be highly correlated; and samples must have been collected from upper soil profiles (<30 cm depth) because most measurable gamma rays emanate from the upper soil foundation (Wong & Harper, 1999). Soil samples were taken within the A and B horizons of the vertical soil profile at each site (where *N* = 895 and 867 sites per horizon, respectively). Because sampling depth for each horizon varied somewhat between sites, average values were calculated for each site. Data for these two horizons were modeled separately.

The secondary dataset was provided by the Department of Environment, Land Water and Planning (hereafter “DELWP test dataset”). These data from 398 locations were originally collected by White et al. (2003) to investigate native vegetation associations in northwestern Victoria. Soil samples were collected by auger boring at 5 and 30 cm deep at each site (note, 30‐cm data were missing from two sites). This dataset was used for independent model evaluation. All soil variables are detailed in Table 1.

**Table 1:** Soil and environmental variables used in modeling for both the primary “VSIS training dataset” and the “DELWP test dataset.”

	Training		Test	
Radiometric variables				
γK (%)^a^	−0.32 to 4.13		−0.28 to 2.83	
γTh (ppm)^a^	−2.11 to 27.9		−3.16 to 19.2	
Soil profile (horizon or depth (cm))	A	B	5	30
Soil texture variables^b^				
Sand (%)	27–94 (66)	27–94 (41)	27–94 (69)	27–94 (59)
Clay (%)	4–57 (24)	4–57 (44)	4–57 (22)	4–57 (30)
Soil chemical variables				
pH	4.3–9.8	4.6–10.2	5–9.4	4.8–9.8
EC (dS/m)	0–86	0–44.7	0.05–8.2	0.05–11
Exchangeable Ca (meq/100 g)	0–36 (55%)	0–32 (70%)	N/A	N/A
Exchangeable K (meq/100 g)	0–4.2 (55%)	0.1–5.1 (70%)	N/A	N/A
Exchangeable Mg (meq/100 g)	0–21 (55%)	0.8–30 (70%)	N/A	N/A
Exchangeable Na (meq/100 g)	0–24 (55%)	0–22 (70%)	N/A	N/A
Chloride (mg/kg)	0–18 (32%)	0–2.4 (53%)	N/A	N/A
Organic carbon (%)	0–99 (48%)	0.1–2.5 (18%)	N/A	N/A
Available water capacity (AWC %)	0–54 (35%)	2–31 (36%)	N/A	N/A
Climate and environmental variables				
Annual radiation (MJ/m^²^/day × 10)^c^	151–184		165–185	
Annual precipitation (mm)^c^	261–900		259–518	
Annual temperature (°C × 10)^c^	106–166		138–167	
Topographic wetness index (TWI)^d^	6341–10365		7202–10343	

a Derived from 50‐m gridded rasters, Department of Economic Development, Jobs, Transport and Resources, Victoria, for airborne gamma radiometric spectrometry surveys.

b Estimate from field texture following Minasny et al. (2007).

c Derived from maps computed from a 50‐m digital elevation model (DEM) using the software package ANUCLIM 5.1 (Houlder, Hutchinson, Nix, & McMahon, 2000). The variables annual radiation and annual temperature had been premultiplied by 10.

d Topographic wetness index computed using the Shuttle Radar Topography Mission (SRTM) 100‐m digital elevation model and TOPOCROP version 1.2 (Schmidt, 2002) with an extension for ArcView 3.2 that implements various Terrain Indices.

Minimum and maximum values are shown, and mean values for sand and clay (%) are given in parentheses. Percent of data available for each variable is indicated in parentheses where dataset was incomplete; N/A indicates data not available. Soil sampling locations are indicated, where VSIS sampling was stratified by A and B horizons (*n* = 895 and 867 sites, respectively) and DELWP sampling was stratified by depth 5 or 30 cm (398 and 396 sites, respectively).

### 2.3 Soil texture variables

While both datasets included field estimates of soil texture class (McDonald et al., 1990) for all sites, laboratory analyses of soil particle fractions were only available for a subset of 209 of the 925 sites in the “VSIS training dataset.” This “VSIS subset” dataset comprised particle size analysis (PSA) for the A horizon at 189 sites and B horizon at 118 sites. In lieu of PSA data for the full VSIS dataset and the “DELWP test dataset,” we predicted mean soil particle fraction from field estimates for the remaining sites (in both VSIS and DELWP datasets) following Minasny et al. (2007). Minasny et al. modeled the relationship between field soil classification and laboratory PSA. We used their model to transform the categorical field texture classes into two new numeric variables: sand (%) and clay (%). We chose to omit silt (%) from our analysis because it is the more difficult to estimate in the field, it is less common in the study area, and sand, clay, and silt components sum to 100, so it was superfluous to model all three components. Sites that had multiple texture entries within a soil horizon (such as A1, A21, and A22) were first transformed into numerical values and then averaged.

Field texture assessment is subjective and can be influenced strongly by other soil properties such as organic matter content (Minasny et al., 2007). We tested for a linear relationship between predicted field texture variables and laboratory PSA (for the VSIS subset dataset) to ascertain whether the relationship between the two variables was reasonable. In a regression of transformed field texture estimates versus laboratory PSA measurements, points located above a 1:1 trend line would indicate field measurements underestimate the true particle size, whereas points below the line would indicate overestimation.

### 2.4 Gamma radiometric data and environmental variables

Spatially explicit independent data for all soil pit and auger locations were extracted from relevant databases. This included gamma radiometric data (Minty, Franklin, Milligan, Richardson, & Wilford, 2009) and modeled terrain and climate data. Further details of soil radiometric and environmental variables are provided in Table 1.

The airborne radiometric survey records the data in units of count per second; however, the available dataset consists of data on transformed scales (% for K and parts per million for Th) intended to reflect ground‐level abundance in a way that is comparable across the land surface. Artifacts such as negative % K and ppm Th values can occur where site detection measures fall below background radiation levels (Minty et al., 2009). Radiometric U signal was not extracted because it generally existed as small traces with high background variation.

### 2.5 Statistical modelling

We had two distinct purposes for modeling the “VSIS training dataset.” First, we aimed to determine what physical soil properties explained variation in either ɣTh or ɣK signals (i.e., explanatory modeling). Second, we aimed to understand how well ɣTh and ɣK plus environmental variables predict the fraction of sand and clay in soils (i.e., predictive modeling). These are typical regression modeling problems. We used boosted regression tree (BRT) models for both tasks because they can reveal relevant relationships and have a demonstrated capability for reliable variable selection, automatic detection of interactions, and robust fitting of trends (Hastie, Tibshirani, and Friedman (2009). BRTs are a form of regression modeling from the machine‐learning discipline that use boosting to combine many simple regression trees to improve predictive performance, and their value has been previously demonstrated in ecological studies (Buston & Elith, 2011; Elith, Leathwick, & Hastie, 2008; Fabricius & De'ath, 2008; Read et al., 2008).

A cross‐validation procedure was used for training and testing both the explanatory and predictive models of the “VSIS training dataset” and to develop models that fit the main trends in the data but would remain general enough to predict well. We used 10‐fold cross‐validation to identify the best tree with minimum predictive error (i.e., minimum error of predictions to new samples), where the data were split in ten “folds” and the model built from nine and validated against the tenth, ten times. Predictive performance was indicated by the “percent deviance explained” for the independent or held‐out data in each iteration of the model.

We used field‐estimated soil particle fraction (predicted from Minasny et al., 2007) for all boosted regression tree models, rather than PSA of soil texture, because we only had 209 observations of PSA compared to 925 field observations for the “VSIS training dataset.” In all cases, predictive deviance was lower for the subset compared to the full dataset.

All BRT models in this study were fitted in R (version 2.12.2, R Core Development Team 2011), using “gbm” package version 1.6‐3.1 (Ridgeway, 2010) plus custom code written by Elith et al. (2008). We used a slow learning rate of 0.005 and a tree size of 3. These settings allowed for reliable estimation of relationships and sufficient complexity to model potential interactions between variables. All response variables were modeled as Gaussian following log transformation of ɣTh, square‐root transformation of ɣK, and logit transformation of sand and clay content (i.e., logit ((y*0.998)+0.001)) to normalize response data.

### 2.6 Independent dataset for model evaluation

We used the secondary “DELWP test dataset” from the same study region to evaluate predictive performance of models developed from the “VSIS training dataset.” We were particularly interested in how well ɣTh and ɣK plus environmental variables can predict soil fractions in an independent dataset. This provides a strong test of the predictive power of our original predictive models of sand and clay fraction, because the datasets were independent, and because the two datasets were collected under projects with nature conservation (DELWP) and agriculture and economic development (VSIS) objectives, respectively.

To predict to the independent dataset, we used model objects from our predictive models (where the response was sand or clay fraction in the upper soil horizon) and performed an external validation procedure on the independent DELWP dataset, following Elith et al. (2008). While the mean and quartiles of the upper soil profiles were equivalent between the datasets, mean sand and clay fraction of the lower soil profile (30 cm depth) of the DELWP dataset were 144% and 68% of mean sand and clay fraction of the B horizon in the VSIS dataset, respectively (Table 1). This was likely because soil samples in the DELWP dataset were taken at specific depths rather than by horizons. While a depth of 5 cm would capture the A horizon most of the time, a depth of 30 cm could sample A, B, and/or C horizon. We therefore decided to restrict external model validation with the DELWP dataset to the upper (5 cm) profile only.

## 3 RESULTS

### 3.1 Comparison between transformed field texture variables and PSA

Transformed field texture measurements estimated sand and clay particle fractions, as measured by laboratory particle size analysis (PSA), with *R* ^2^ = 0.64 for relationship between field‐estimated clay and PSA and *R* ^2^ = 0.67 for sand. Linear relationships between the transformed field estimates and laboratory particle size measurements (i.e., PSA) for the two soil texture variables in the “VSIS subset dataset” were very close to the ideal 1:1 lines, with relatively narrow confidence intervals (Fig. S1).

However, the range of field‐estimated clay and sand fractions for the “VSIS training dataset” were restricted compared to particle size analysis (PSA) measures from the same dataset (Fig. S1). Maximum field‐estimated clay content was 60%, compared to ~20 measures of PSA between 60% and 80%, while field‐estimated sand had a minimum content of ~25% compared to >20 measures of PSA between 10 and 20.

### 3.2 Explanatory models for radiometric K and Th

**Figure 2:**
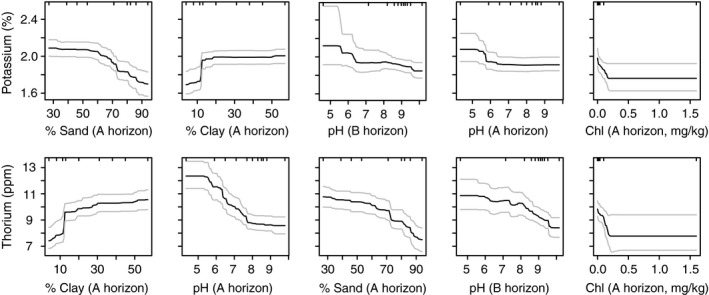
Partial dependence plots of five most influential variables in explanatory boosted regression tree (BRT) models for potassium (ɣK %) and thorium (ɣTh ppm) radiometric data (from “VSIS training dataset”). A and B indicate upper and lower soil horizons, respectively. The model for ɣK had a predictive deviance of 36%, and variable contribution to the final model was as follows: sand % (A, 22.0%), clay % (A, 18.3%), soil pH (A and B, 8.7% and 6.2%, respectively), and soil chloride mg/kg (A, 5.5%). The model for ɣTh had predictive deviance of 53%, and variable contribution to final model was as follows: clay % (A, 20.8%), pH (A, 19.1%), sand % (A, 18.6%), pH (B, 7.8%), and chloride mg/kg (A, 4.6%). NB. *Y*‐axis is plotted in the original scale for γK and γTh, but models were run on square‐root‐transformed data for γK and log‐transformed data for γTh. Gray lines show 95% confidence intervals

Boosted regression tree models explained 53% and 36% of cross‐validation deviance for ɣTh and ɣK, respectively, in the “VSIS training dataset” (Figure 2). Sand and clay fraction (%) were generally the most influential variables in both models with sand explaining 22.0% of ɣK and 18.6% of ɣTh, and clay explaining 18.3% of ɣK and 20.8% of ɣTh. Other influential variables in the both final models were soil pH (horizons A and B) and soil chloride mg/kg (horizon A) and AWC. Results from explanatory models of the smaller “DELWP test dataset” were consistent with models of the “VSIS training dataset” (see Table S1 for further information).

### 3.3 Predictive models for soil texture variables

**Figure 3:**
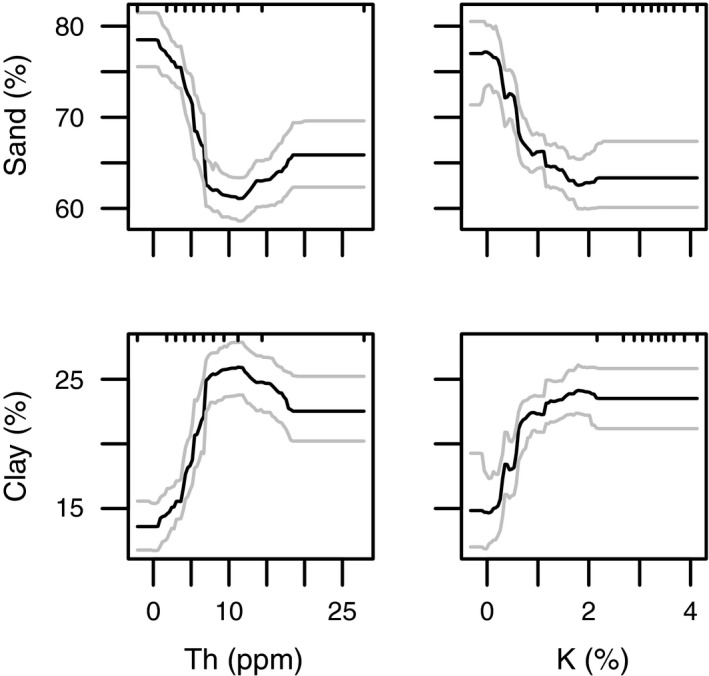
Partial dependence plots of percent sand and clay (A horizon) from predictive boosted regression tree (BRT) models of “VSIS training dataset.” Plots show the influence of radiometric variables potassium (ɣK %) and thorium (ɣTh ppm). Soil texture percentage was transformed from field‐estimated soil texture classes following Minasny et al. (2007). Gray lines show 95% confidence intervals. For details on variable relative influence, refer to Table 2. Note, plots show back‐transformed response variables; the original models were run on logit‐transformed data

Soil radiometric data, combined with environmental data, successfully predicted soil texture in the upper A horizon, using boosted regression tree models (Table 2, Figure 3). Cross‐validation of models with held‐out data gave predictive deviances of ~43% for both sand and clay fractions, respectively. External validation with the independent DELWP dataset showed weaker predictive power with deviances of ~30% for both sand and clay fractions, respectively.

**Table 2:** Predictive boosted regression tree (BRT) models of field‐estimated sand (%) and clay (%) in the upper (A) and lower (B) soil profiles for the “VSIS training datasets,” showing relative influence (%) of model variables: gamma radiometric (ɣTh and ɣK count) data, topographic wetness index (TWI), and climate data

	Sand		Clay	
	A	B	A	B
Relative influence				
γTh	44.7	42.5	45.0	40.2
γK	26.0	8.2	25.5	8.2
TWI	16.1	10.8	16.8	12.0
Annual precipitation	9.1	26.6	8.6	27.4
Annual temperature	2.6	4.6	2.6	4.0
Annual radiation	1.4	7.3	1.3	8.1
Predictive deviance				
Internal cv on held‐out data	43.0	29.2	43.2	29.8
External validation on “DELWP test dataset”	30.0	–	30.25	–

Predictive deviance (%) of the BRT model was calculated by internal cross‐validation on held‐out VSIS training data and external validation on the independent “DELWP test dataset” (where the upper profile (A) is depth 5 cm and lower (B) is depth 30 cm). Sand and clay content were classified in the field, transformed to percent following Minasny et al. (2007), and logit‐transformed (logit ((y*0.998)+0.001)) prior to analyses.

Both ɣTh and ɣK were the most important predictors of soil texture (A horizon) in predictive BRT models (Table 2). ɣTh had relative influence of ~45% on both sand and clay fractions, while ɣK had less relative influence of ~26% on both fractions. Sand (%) was negatively and clay was positively related to radiometric counts (ɣTh and ɣK), with similar response shapes for both types of gamma‐ray emissions: a steep slope for low levels of emissions followed by a plateau at 10 for ɣTh and 2 for ɣK (Figure 3).

### 3.4 External model validation

**Figure 4:**
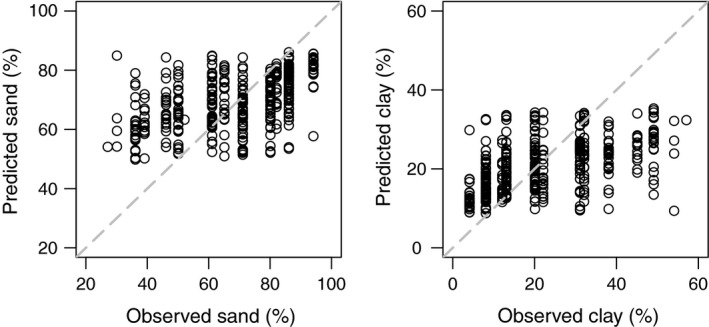
Plot of observed A‐horizon soil fractions in “DELWP test data” versus predicted soil fractions derived from boosted regression tree (BRT) model of “VSIS training data.” Observed soil fractions were from field‐estimated sand and clay content, transformed to % sand and clay, and predicted soil fractions were from the VSIS (training) model based on field estimate of soil sand and clay content, transformed to % sand and clay. Transformations followed Minasny et al. (2007)

The range of soil fractions predicted by our BRT models for the independent DELWP test dataset were narrower than those observed (Figure 4). Predicted clay fraction at 5 cm depth ranged from ~9 to 35 compared to the observed range of 4%–57%, while predicted sand fraction at 5 cm depth ranged from ~50 to 86 compared to the observed range of 27%–94%.

## 4 DISCUSSION

Airborne radiometric data are readily available (Minty et al., 2009), accurate, and continuously scaled and show potential as a surrogate of soil texture for use in plant and vegetation modeling. Our study demonstrates that airborne gamma radiometric signals of potassium and thorium (ɣK and ɣTh) were moderately explained by soil physical variables within the context of our 40,000‐km^2^ study region and that in turn K and Th could be useful predictors of soil texture at that scale. The results of this study, together with the underlying theory and mechanistic explanations from the literature, provide qualified support for the use of radiometric data as surrogates of soil texture in the study region.

### 4.1 Effects of soil texture on radiometric signals

The most important soil physical properties explaining variation in the gamma radiometric data in northwestern Victoria were soil sand and clay fractions in the A horizon. We found that gamma radiometric Th and K both had strong positive relationships with clay (%) and negative relationships with sand (%). This result conforms with the expectation, and earlier studies relating soil properties and proximal sensing of gamma emissions (e.g., Coulouma et al., 2016). The relationship with clay and sand likely reflects the relationship between soil texture and cation exchange capacity (CEC). While CEC may vary with parent material and degree of weathering (Wilford & Minty, 2006), soils with a higher clay fraction tend to have a high CEC and soils with a high sand fraction tend to have a low to negligible CEC (Donahue, Miller, & Shickluna, 1977). Pure sand has no electrical charge and a low specific surface area, whereas clay minerals have surfaces that have negatively charged sites, which adsorb and retain positively charged ions (including metallic ions of thorium and potassium) and have a high specific surface area (Ellis, 1987; Leonte, Nott, & Dunsmuir, 2003; Rachkova, Shuktomova, & Taskaev, 2010). In similar orographic formations in New South Wales, Cattle et al. (2003) confirmed that gamma radiometric data moderately explained soil composition, particularly clay and silt enrichment of topsoils.

While these results generally vindicate the use of gamma radiometric data as a coarse surrogate for soil texture in previous studies (White et al., 2003; Duncan et al., 2007; Vesk et al., 2010; Read et al., 2008; 2011), considerable residual variation and prediction error remained from our model validation procedures. This residual variation could be due to the nature of our investigation, which integrated independent and variously complete datasets over a considerable geographic scale with a wide range of pedogenic and geomorphological processes.

### 4.2 Sources of error and the predictive performance of models

The performance of boosted regression tree (BRT) models predicting sand and clay content was higher when predicting to held‐out data (with internal cross‐validation) than to the independent “DELWP test dataset” (Table 2). This reduced performance predicting to an independent dataset is unsurprising given distinct purposes of sampling between the two datasets and that their soil survey methodology was not identical. The “VSIS training dataset” was collected over a thirty‐year period by a large number of individuals, whereas the DELWP dataset was collected during a targeted survey by a small team of dedicated fieldworkers. Also, soil sampling was stratified into A and B horizons for the VSIS dataset, whereas soil sampling was stratified by depth for the DELWP dataset. Both these factors would contribute to the reduced model performance when predicting to the independent DELWP dataset.

While the strong positive relationships between PSA and field‐estimated soil texture (Fig. S1) support the utility of Minasny et al.'s (2007) conversion table for transforming texture classes into continuous variables, our data suggest this transformation is most useful for calculating soil sand and clay fraction at moderate levels. Field estimates of soil texture tended to offer poor detection of sand content <25% and clay content >60%. Overall, field studies appeared to underestimate clay and overestimate sand content, with plots of field‐estimated texture vs laboratory PSA (Fig. S1) showing linear trend lines above the 1:1 for clay and below 1:1 for sand.

Considering the error related to field‐estimated texture data, we believe our models provide strong support for the utility of radiometric data as a surrogate of soil texture. Equivalent models developed with PSA data would likely show a higher predictive performance, but our PSA dataset included <30% of the number of observations of the field‐estimated dataset, so we did not detect improved predictive performance of BRT models developed with PSA data.

### 4.3 Effects of pH on radiometric signals

Gamma K and Th data were related to soil pH, with stronger radiometric signals on acidic soils (Figure 2; Table 2). Dierke and Werban (2013) also found a negative relationship between Th and pH in a high‐resolution study of one experimental site with homogenous texture. They observed that the relationship was only applicable up to pH of 7. Our models suggest a similarly constrained relationship between K and pH, but for Th, the negative relationship was sustained up to 8 for the A‐horizon sample and up to the maximum pH sampled for K in the B horizon. By contrast, Wong and Harper (1999) found a strong log‐linear relationship between ground‐based measured ɣK data and soil pH, but they observed stronger ɣK signals with more *alkaline* soil. They concluded the relationship was spurious. Together, results indicate potential relationships between gamma radiation and soil pH exist, but suggest the relationships are not simple and may vary with study area.

### 4.4 Limitations on the gamma radiation—soil texture relationship and broader application

The geomorphology of our study region is expected to be a relatively favorable landscape for relating gamma radiometric signal to soil properties, being mostly comprised of well‐sorted surficial materials deposited by wind and water and with virtually no outcroppings of groundwater and bedrock. Even so, Wilford, Bierwirth, and Craig (1997) noted interpretation of radiometric data could be complicated where parent materials of those wind‐ and water‐transported sediments have different origins. For example, Cattle et al.'s (2003) 10 detailed case studies of ground‐based and airborne gamma radiometric data and associated soil formation narratives, found that superficially similar particles may be K‐enriched or K‐depleted depending on the parent material, and thus emit considerably different levels of gamma radiation. Their work suggests a natural limit on a soil textural interpretation of soil radiometric data within our study area and invites further exploration of radiometric data and soil properties with more tightly coupled datasets over similarly extensive landscapes.

The degree to which the relationships identified in our study area could be extrapolated to other regions has not been well tested. Researchers in similarly extensive and relatively uniform sedimentary landscapes could have some optimism, and anticipate a better result from the incorporation of contemporaneous, and uniformly collected soil site data. For other landscape types, there is good reason to be circumspect: Mixtures of sedimentary, plutonic, and volcanic geology; outcropping rock; greater relief; and variable microtopography would all individually be expected to increase the difficulty of modeling soil texture from radiometric data, to say nothing of their combination.

### 4.5 Some lessons from proximal sensing

Ecologists interested in the prospects of reliably modeling soil texture from remotely sensed sources should also heed the insights emerging from studies using proximally sensed radiometric data, which permit tighter linking of soil properties and emissions. For example, Priori et al. (2014) predicted soil clay and sand content relatively well across three pedogenic groupings but found that surface stony elements significantly complicated proximal gamma radiation signal, either impeding emissions (calcareous stones) or contributing to strong emissions in the case of materials with high radionuclide content. Nonetheless, using proximally sensed radiometric data, Heggeman et al. (2017) recently obtained good predictive performance of soil texture across a heterogeneous set of 10 pedogenically distinct sites using supervised, nonlinear machine‐learning modeling techniques. While their example is based on intensive replication within relatively few sites, it is encouraging.

Given the local idiosyncrasies of soil landscapes, other inherently interpolative spatial modeling approaches used in geostatistics such as such as regression kriging, multiadaptive regression splines, and multivariate thin‐plate splines (Hutchinson and Gessler 1993; Omuto & Vargas, 2014; Ballabio, Panagos, & Monatanarella, 2016) may be better suited to transforming radiometric data and soil pit data into spatially explicit models of soil properties. Moreover, Gray, Bishop, and Wilford (2016) recently noted that the effective modeling of soil properties awaits useful covariates that represent parent material. Presumably, such developments will greatly improve the utility of airborne radiometric data for modeling soil properties in landscapes and contexts such as ours, and beyond.

## 5 CONCLUSION

Plant ecologists will be encouraged to know there is a remote sensing product that, in particular geomorphological contexts, is informative at broad scales about the soil properties most commonly related to vegetation associations. Our study demonstrated that radiometric data are a promising, although qualified, surrogate for soil texture. Relationships still need to be built and validated for a wider range of soil types and pedogenic and geomorphological contexts, probably combining and contrasting remote and proximally sensed gamma‐ray emission data, but we are optimistic that useful tools for improving predictive power of plant distribution and dynamic landscape vegetation models are not far away.

## AUTHOR CONTRIBUTIONS

PV, MW, and DD conceived of the study, and the design was further elaborated with input from CH. MW led primary data collection for the DELWP test dataset, while CH managed the integration of the various datasets. The specific analyses presented were undertaken by CR and CH, and all authors contributed to their interpretation. The article was drafted by CR and CH and was critically reviewed by DD, PV, and MW. All authors consented to the publication of the final version.
