# Supplementary figures and images for: Useful surrogates of soil texture for plant ecologists from airborne gamma‐ray detection
Source: Ecol Evol. 2018 Jan 16;8(4):1974–83. doi: 10.1002/ece3.3417 (PMC5817144; doi:10.1002/ece3.3417)

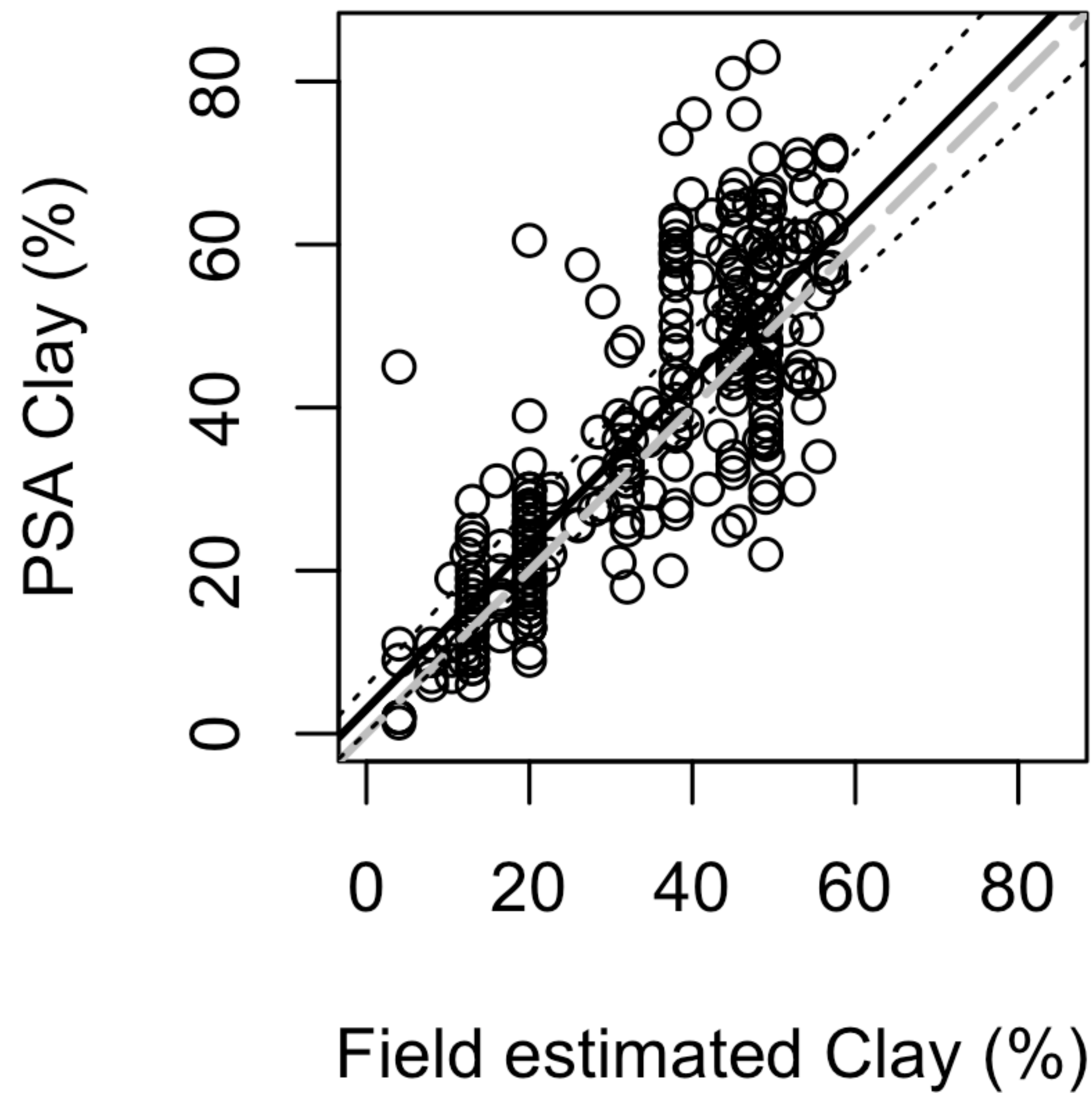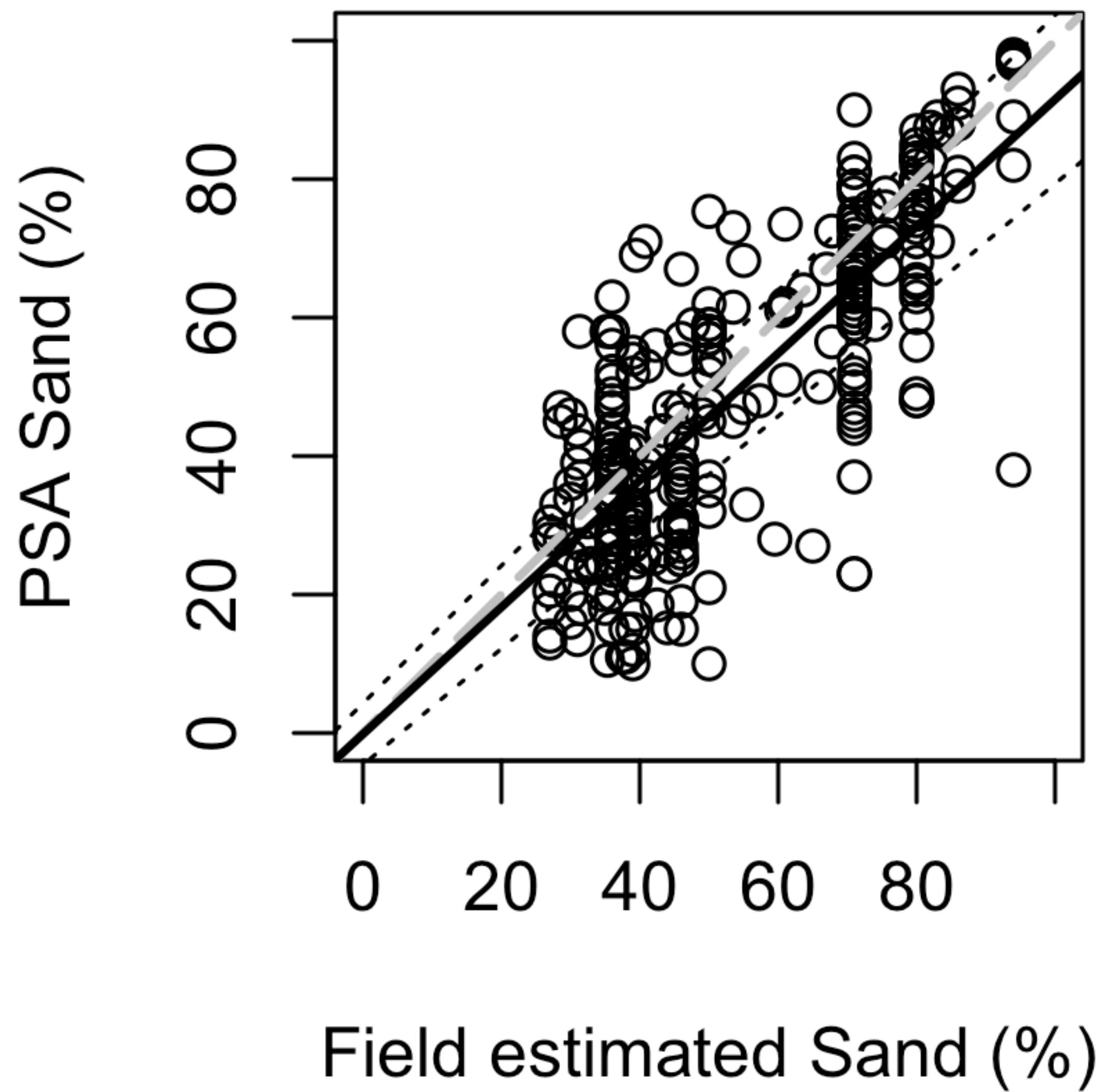

Supplement: Supplementary file 1 [file ECE3-8-1974-s001.pdf]
