# Supplementary material for: Useful surrogates of soil texture for plant ecologists from airborne gamma‐ray detection
Source: Ecol Evol. 2018 Jan 16;8(4):1974–83. doi: 10.1002/ece3.3417 (PMC5817144; doi:10.1002/ece3.3417)
Supplement: Supplementary file 2 [file ECE3-8-1974-s002.docx]

**SupplementaryTable 1.** Explanatory boosted regression tree models of ɣTh and ɣK for DELWP (Test) dataset. Predictive deviance was calculated using internal cross-validation with held-out data.

| *Relative influence* | Sand | | Clay | | |
| --- | --- | --- | --- | --- | --- |
|  | 5 cm | 30 cm | | 5 cm | 30 cm |
| γTh | 33.4 | 53.3 | | 35.4 | 55.0 |
| γK | 30.5 | 10.0 | | 28.2 | 8.8 |
| Total wetness index | 12.6 | 6.9 | | 12.7 | 7.0 |
| Annual Precipitation | 13.9 | 15.5 | | 15.1 | 15.4 |
| Annual Temperature | 6.1 | 5.0 | | 5.3 | 4.6 |
| Annual Radiation | 3.5 | 9.1 | | 3.4 | 9.2 |
| *Predictive deviance* | 35.7 | 43.1 | | 38.6 | 43.68 |
